# Foveation Pipeline for 360° Video-Based Telemedicine

**DOI:** 10.3390/s20082264

**Published:** 2020-04-16

**Authors:** Muhammad Firdaus Syawaludin, Myungho Lee, Jae-In Hwang

**Affiliations:** Imaging Media Research Center, Korea Institute of Science and Technology (KIST), Seoul 02792, Korea; firdaus.lubis@kist.re.kr (M.F.S.); mlee@kist.re.kr (M.L.)

**Keywords:** HMD, telemedicine, foveation, multi-resolution

## Abstract

Pan-tilt-zoom (PTZ) and omnidirectional cameras serve as a video-mediated communication interface for telemedicine. Most cases use either PTZ or omnidirectional cameras exclusively; even when used together, images from the two are shown separately on 2D displays. Conventional foveated imaging techniques may offer a solution for exploiting the benefits of both cameras, i.e., the high resolution of the PTZ camera and the wide field-of-view of the omnidirectional camera, but displaying the unified image on a 2D display would reduce the benefit of “omni-” directionality. In this paper, we introduce a foveated imaging pipeline designed to support virtual reality head-mounted displays (HMDs). The pipeline consists of two parallel processes: one for estimating parameters for the integration of the two images and another for rendering images in real time. A control mechanism for placing the foveal region (i.e., high-resolution area) in the scene and zooming is also proposed. Our evaluations showed that the proposed pipeline achieved, on average, 17 frames per second when rendering the foveated view on an HMD, and showed angular resolution improvement on the foveal region compared with the omnidirectional camera view. However, the improvement was less significant when the zoom level was 8× and more. We discuss possible improvement points and future research directions.

## 1. Introduction

Telemedicine is broadly defined as information and communications technologies that provide and support health care between participants in the distance [1]. From a simple voice communication to a teleoperated surgical robot, telemedicine includes a wide range of different technologies. Nonetheless, most systems are based on video-mediated communication, through which social interaction between two parties as well as information acquisition takes place. Humanoid robots or virtual reality (VR) technologies have been employed to improve the social interaction aspects of telemedicine, and various cameras and image processing techniques have been applied for visual information acquisition.

One of the widely used camera types is a pan-tilt-zoom (PTZ) camera [2]. PTZ refers to the camera’s ability to move side-by-side (panning), up-and-down (tilting), and enlarge/shrink the captured scene (zooming). This ability allowed the remote physician to control the camera orientation to communicate with in situ medical practitioners with regards to the context, and zoom in when an up-close view is needed, e.g., inspecting wounds or reading medical instrument. However, the PTZ camera could be difficult to control. Chapman et al. [3] designed an ambulance-based telestroke platform with a PTZ camera and assessed its usability. In their evaluation, one physician commented on how difficult it was to maneuver the PTZ camera.

Another popular camera used in telemedicine is an omnidirectional camera. Unlike PTZ cameras, omnidirectional cameras capture a semi- or entire spherical visual field, thus there is no need for mechanical maneuvering of the view direction. Laetitia et al. [4] used an omnidirectional camera in a pre-hospital telemedicine scenario. The camera allowed a remote physician to see the entire scene inside an ambulance, including a patient and first responders. Also, the camera supported digital zooming: an image processing technique to enlarge certain areas on the image. Though an omnidirectional camera could capture the entire scene, it did not provide better resolution compared with the PTZ camera. Digital zooming would only enlarge the image region pixel size (with probably additional image processing technique) without changing the image angular resolution. The angular resolution was certainly much lower compared with the PTZ camera which could do optical zooming.

The problem of increasing FOV while providing high angular resolution around the region of interest (ROI) has been the primary issue of foveated imaging research. The research field takes advantage of the foveation phenomenon in the human visual system (HVS) caused by the non-uniform distribution of photoreceptor cells (rods and cones) that lines the retina. Cones are less sensitive to light compared with rods but provide color perception and perceive finer details in the image. Thus, visual acuity, e.g., spatial resolution, of the human eye will be highest in the region that contains a high concentration of cones. The cone density is highest at the fovea and drops off sharply as the distance from the fovea increases. Consequently, the visual acuity is highest within a range of 2${}^{\circ }$ from the fovea and falls off very sharply beyond that [5].

Two approaches exist when capturing a scene for foveated imaging [6]. First, specialized imaging sensors have been designed and applied to capture the scene with different resolutions. Examples are non-uniform sampling sensors [7], foveated optical distortion of the lens [8], and multiple-channel segmented single sensors [9]. Second, two or more cameras can be used and post-processed to generate a unified foveated image [9,10]. Though custom imaging sensors have been proposed [9,11], this approach also exploits off-the-shelf cameras [10]. We follow the latter approach, in agreement with Carles et al. [6]’s argument that the low-cost, high-performance computing has made post-processing an appealing solution compared to the complexity of custom sensor and optics design.

In this paper, we present a foveated imaging pipeline that integrates a wide-FOV image from an omnidirectional camera with a high-resolution ROI image from a PTZ camera. Unlike most foveated imaging research for wide FOV, we specifically designed our pipeline to support VR HMD, thereby maximizing the benefits of the use of omnidirectional camera—i.e., head synced view control and consequent increased spatial awareness [12]. To do so, we separated our pipeline into two parallel stages: modeling and rendering. Instead of integrating two images in pixel level, we adjusted the positions of 3D geometric primitives in the modeling stage and rendered the camera images onto the corresponding primitives in the rendering stage. The positions of the primitives are updated only when the ROI (i.e., PTZ orientation) is changed. Regardless of the modeling stage, the rendering stage keeps updating frames from each camera. Thus, the computational load of the modeling stage does not affect the frame rate of the foveated view. Moreover, we applied a zoom-based adjustment and blend mask for the seamless integration of the two images.

**Figure 1:**
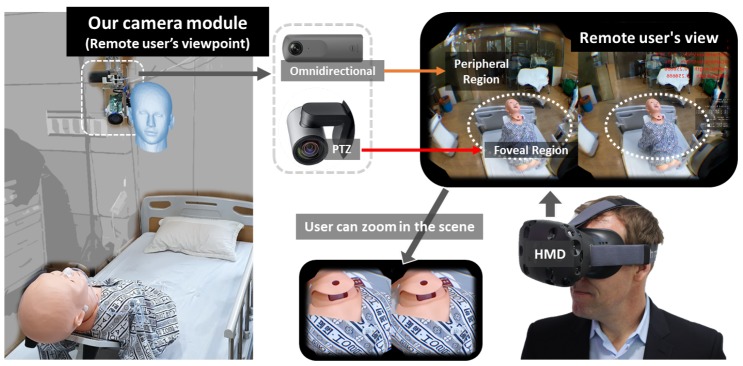
Overview of our proposed system.

The telemedicine system we develop with our foveated imaging pipeline is shown in Figure 1. Our camera module consists of an omnidirectional and PTZ camera pair, and a remote physician sees the scene from the position of the camera module through a VR HMD. The foveal region (ROI) contains the target object (the patient). The foveal region is rendered in high angular resolution and smoothly superimposed onto the peripheral region, i.e., 360${}^{\circ }$ background. The remote physician can zoom in the scene to inspect the wound area in detail.

Our contributions are summarized as follows: First, we propose a telemedicine system consisting of an omnidirectional and high-resolution PTZ cameras that supports 360${}^{\circ }$ video streaming for VR HMDs with a high angular resolution for the foveal region. Moreover, our system allows the remote user to zoom in the foveal region in high detail. Second, we present a novel foveated imaging pipeline for VR HMDs, which includes parallel modeling and rendering stages. In that, additional techniques, such as zoom-level adjustment and masking, are also devised to improve the quality of the foveated image.

The rest of the paper is organized as follows: In Section 2, we provide a literature review of the foveated imaging system. Section 3 details our hybrid camera module and the proposed pipeline that enables the real-time rendering of the foveated view on HMDs. In Section 4, we present evaluations on the proposed system, in terms of the angular resolution for the foveal region, overall processing time, and frame per second achieved. Then, we further discuss improvement points based on the results of our evaluations. Finally, we summarize and conclude the paper in Section 5.

## 2. Related Works

Foveated imaging research not only addresses how to design the cameras but also how to use or view the foveated image itself. One of the early uses of such foveated imaging-based camera systems was for object tracking, whether stationary [13], on a robot [10], or mobile agent-based surveillance [14]. Typically, an omnidirectional image is processed with a fast algorithm to extract the ROI. The centroid of the ROI combined with external camera parameters is then converted to the pan and tilt angle of the PTZ camera. The PTZ camera then either shows the object in a higher resolution or further classifies it. In this research, they analyze the obtained image separately. The way of how they present the images is not essential.

On the other hand, several applications require more attention on how they should present both images to the user. Qin et al. [15] designed a laparoscope that consists of two fully integrated imaging probes: a wide-angle and a high-magnification probe. They showed the wide-FOV and high-resolution images in separate windows. However, the separation between the two views will increase the user workload when navigating as they need to split the focus between the two windows [16].

Several researchers have also tried to integrate the multi-resolution images that come from a single PTZ camera [17] or a pair of wide-angle non-full panoramic 360${}^{\circ }$ camera and PTZ camera [18,19] for surveillance application. The problem of integrating the different resolution images is located on how we would preserve the high-resolution region detail. Precisely, the common way in mosaicking different images are by performing an image stitching process in pixel level during graphics rendering. However, merging the high-resolution image onto a low-resolution one will certainly reduce the detail captured in a stitched high-resolution region. The foveal region on foveated images will not have the same high angular resolution to the PTZ camera, even if we try to digitally zoom in on the foveal region. On the contrary, merging the low-resolution image onto the high-resolution one will require the upsampling of the low resolution, thus make the target rendered image bigger than the original [19]. Existing solutions to preserve the high-resolution detail is either to construct a 3D model of the scene [17] or use a large high-resolution display [18]. Another way is to use a separate output display to render the output of each camera [20]. However, these viewing approaches are impractical to implement for our application. Their resulting images cannot be rendered directly for VR HMDs.

Other researchers are attempting to take advantage of foveation phenomena in panoramic 360${}^{\circ }$ video streaming. However, the purpose of the foveation is to save the streaming bandwidth and computational power [21]. Thus, instead of trying to improve the quality of the 360${}^{\circ }$ on the foveal region, they reduce the resolution rendered in the peripheral region. This gives a direct implication on the hardware design of the camera they used. For example, they use a camera rig containing several high-resolution cameras to capture 360${}^{\circ }$ scene and then locally stitch the images in the server [22,23]. They decided which region is rendered in high resolution based on some criteria, such as user eye-gaze orientation [23] or saliency importance of each region [22,23]. They then render foveal regions at a higher resolution and peripheral region in a lower resolution on pixel level [23]. Alternatively, they perform a non-uniform spherical sampling ray approach and adjust the graphics vertices position accordingly to allocate more pixels on the foveal regions [22]. However, aside far from real-time performance, the approach also distorts the foveal-peripheral region boundary.

We aim to render the foveal region in 360${}^{\circ }$ video stream in high resolution while preserving the bandwidth as efficiently as possible. Inspired by the usage of hybrid camera for surveillance systems, we use an omnidirectional (360${}^{\circ }$ FOV) and PTZ camera pair for 360${}^{\circ }$ streaming on VR HMDs. Compared to other foveated 360${}^{\circ }$ videos [22,23], the usage of the PTZ camera in our system will enable a remote user to see the foveal region up-close in detail with its zoom component. However, it raises additional challenges on how we can preserve the high angular resolution detail for high optical zooming value. High optical zooming PTZ cameras will render only a small part of the captured scene compared to panoramic 360${}^{\circ }$ images captured by the omnidirectional camera. While we can use common direct stitching techniques on a pixel level to render the foveated view on high-resolution 2D monitor, the VR HMD display pixel resolution is not high enough to cover the whole multi-resolution foveated view while preserving the foveal region high angular resolution. Thus, we propose a foveated stitching pipeline that works on the vertices level. Our vertices level stitching method separates the foveal region mesh from that of the peripheral region. The mesh separation allows the foveal region mesh to preserve its texture source (PTZ camera) angular resolution. We also describe the accompanying zooming mechanism so that the local physician can inspect the foveal region in high-quality while zooming in. Table 1 shows a comparison of the previous foveated imaging systems along with ours.

## 3. System Design

In this section, we introduce our hardware design, foveated view, foveated imaging pipeline, and control mechanism separately in each subsection. The hardware design explains the components we used to build a hybrid camera module and the display used. The foveated view subsection briefs the foveated view and the benefit of having such a view. In the foveated imaging pipeline subsection, we explain how we render the foveated view in real time while preserving the high angular resolution of the foveal region. Furthermore, we delineate how we avoid a mismatch between peripheral and foveal region during the zooming process. Finally, the control mechanism subsection explains how a remote physician places the foveal region and uses our system to see the target object in finer detail.

### 3.1. Hardware Design

**Figure 2:**
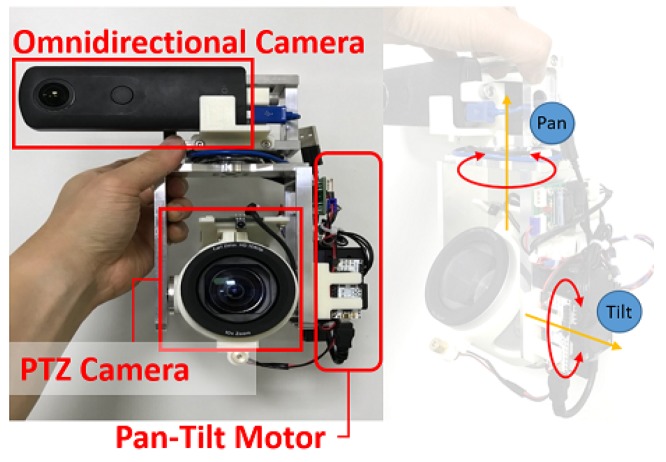
Prototype of the camera module.

**Hybrid Camera Module:** The hybrid camera module was built with aluminum alloy frames, two cameras (an omnidirectional and a PTZ camera), and two external servo actuators (see Figure 2). The servos control the pan and tilt angle of the PTZ camera. We placed the cameras close to each other. We used a RICOH THETA V omnidirectional camera and a Logitech PTZ Pro camera. The RICOH THETA V was connected through a USB 3.0 cable to a local computer and provided live streaming (3840 × 1920, 30 fps) of the surrounding local environment. The Logitech PTZ Pro, which can provide up to 10× optical zoom, was also connected to the local computer and streamed a part of the local environment in its angle at a high-definition (1920 × 1080, 30 fps). The two cameras have different FOVs. The omnidirectional camera captures 360${}^{\circ }$*Horizontal FOV* (*HFOV*) and 180${}^{\circ }$ *Vertical FOV* (*VFOV*) scene in *equirectangular* format. Meanwhile, the PTZ camera captures a 90${}^{\circ }$*Diagonal FOV* (*DFOV*) [24] which corresponds to HFOV = 82.15${}^{\circ }$ and VFOV = 52.24${}^{\circ }$ based on the FOV conversion equation in [25]. We removed the original bracket and motors from the PTZ camera. Instead, we used two Dynamixel XM430-W210-R servos as the external actuators for faster and more accurate pan-tilt controls. By doing this, we achieved 77 RPM and 0.0439${}^{\circ }$/step precision of the pan/tilt angle.

**Display:** We used a VR HMD to display our foveated view, and we additionally implemented a desktop monitor version of the foveated view as well. We used HTC Vive HMD which, by default, has approximately 111${}^{\circ }$ VFOV and 105${}^{\circ }$ HFOV. For each eye, the foveated view is always rendered at 1440 × 1600 pixel resolution size. The head motion parallax in the HMD version was not supported due to the stationary placement of the hybrid camera. For the desktop monitor, the view is always rendered in 1920 × 1080 pixel size. By default, we rendered the foveated view with VFOV = 52${}^{\circ }$. Digital and optical zooming of the foveated view in both versions can be controlled by the user by using our proposed zooming mechanism explained in Section 3.4.2.

### 3.2. Foveated View

**Figure 3:**
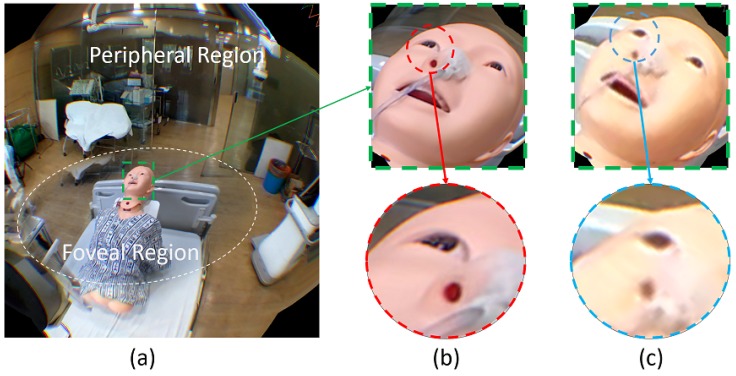
Foveated view consists of foveal and peripheral region (**a**). Zoom-in process of foveated view around target object (**b**) preserves the source (PTZ) angular resolution around eye and nose. Zoom-in process of omnidirectional view (**c**) is shown as comparison. Compared to omnidirectional view, the foveated view can preserve the angular resolution detail around eye and nose.

Our foveated view renders the foveal region at the PTZ camera’s angular resolution while rendering the peripheral region at the omnidirectional camera’s angular resolution. Figure 3a shows the illustration of the foveated view. Please note that Figure 3a showed the perspective projected view rendered on HMD at a certain viewing angle.

The high-resolution foveal region comes from the PTZ camera video stream and the low-resolution peripheral region comes from the omnidirectional camera video stream. We stitch the PTZ camera image onto the omnidirectional camera image, forming the foveated view. Figure 3b,c show the zoomed-in versions of mannequin head in our foveated view and the conventional omnidirectional camera only (omnidirectional view) case respectively. Compared to the omnidirectional view case, our foveated view has a better image quality, which is in line with our quantitative image quality measure present in Section 4.4.

### 3.3. Foveated Imaging Pipeline

**Figure 4:**
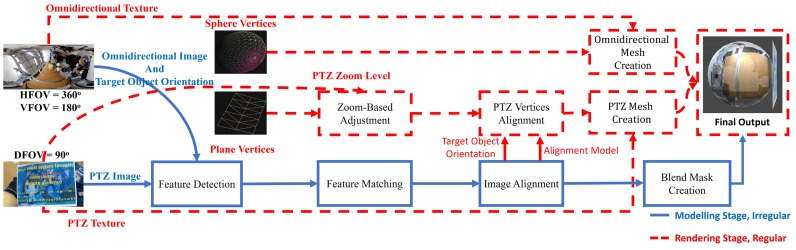
Foveated Imaging pipeline.

Figure 4 shows our foveated imaging pipeline. The key of our pipeline lies in the separation of the two stages (modeling and rendering stage) and the separation of PTZ planar mesh from omnidirectional sphere mesh. The meshes (i.e., PTZ planar and omnidirectional sphere) are formed by the combination of vertices (i.e., PTZ planar and omnidirectional sphere vertices) with texture (i.e., PTZ planar and omnidirectional equirectangular texture). Vertices are a collection of the vertex in 3D graphics that defines information such as the position, normal, and corresponding texture coordinate vector of the objects to be rendered.

Please note that the red and blue color lines and blocks in Figure 4 represent the rendering and modeling stage blocks, respectively. Here, though they are essentially the same, we use the terms *image* and *texture* to distinguish their use in the modeling stage and rendering stage. This separation is to emphasize that we process the omnidirectional and PTZ captured video stream as an image in the modeling stage and treat it as a texture in the rendering stage.

The output of the modeling stage is an alignment model of PTZ planar vertices with respect to omnidirectional sphere vertices and blend mask. The alignment model corresponds to the relative 3D position matrix between PTZ planar and omnidirectional sphere vertices while the blend mask is a 1D image mask used to blend the omnidirectional equirectangular and PTZ planar texture. This stage is triggered when the user intends to examine a certain object, which corresponds to the foveal region update in Section 3.4.1. On the other hand, the rendering stage runs for every frame and renders the PTZ and omnidirectional meshes, resulting in the foveated view. This stage separation allows the rendering stage to run in real time without having to update the alignment model for every frame.

Instead of performing the stitching process of foveal and peripheral region on the pixel level, we perform the stitching process on the vertices level. The illustration of our stitch result can be seen from Figure 4 *Final Output* block. The PTZ planar sphere mesh location will be adjusted according to the alignment model while the omnidirectional sphere mesh location will stay fixed. Both PTZ planar and omnidirectional sphere mesh stays separate after the stitching process. This mesh separation makes the peripheral region of the foveated image have the same angular resolution as the omnidirectional image and the foveal region have the same angular resolution as the PTZ image.

However, even though the proposed pipeline could preserve the foveal region angular resolution the same as PTZ angular resolution, the foveal region on the rendered foveated view will still occupy similar pixel size as the corresponding region on omnidirectional view. For example, if the patient object in Figure 3 occupies 400 × 400 number of pixels in an omnidirectional view, then the patient in the foveal region of foveated view will still be rendered on 400 × 400 number of pixels regardless of the PTZ angular resolution. In other words, the user will not gain the benefit of the angular resolution improvement in the foveal region. Therefore, we also propose the zooming mechanism that can be applied so the user can see the foveal region in a larger view. This zooming mechanism will be explained in Section 3.4.2.

#### 3.3.1. Modeling Stage

The purpose of the modeling stage is to update the alignment model and creating the blend mask. To extract features in the omnidirectional image, we perform perspective projection on omnidirectional image with equirectangular to rectilinear transformation based on current target object orientation [26]. This transformation generates the omnidirectional planar image.

We used SIFT [27] feature detector to extract 2D features in both omnidirectional planar and PTZ image in *Feature Detection* block. In *Feature Matching* block, we adopt a similar RATIO approach used by Lowe et al. [27]. Precisely, for every feature on the PTZ image, we found its two closest neighbors on the omnidirectional planar image with FLANN matcher [28]. We then measured the ratio between the distance of the PTZ features to its first-closest neighbor with the distance of the said PTZ feature to its second-closest neighbor. The correct matches need to have the first-closest neighbor significantly closer than the first-closest incorrect match (i.e., second-closest neighbor) to achieve reliable matching. Thus, we set certain threshold values and picked only feature pairs whose ratio is lower than the predefined threshold. We did not limit the maximum number of feature matches.

In *Image Alignment* block, we used a 2D homography model to find the best homography model that align PTZ image onto omnidirectional planar image [29]. For *Blend Mask Creation* block, we used a predefined ellipse-form blend mask to do alpha blending. In the current implementation, we manually defined the ellipse horizontal and vertical radius. We passed the homography model, target object orientation, and blend mask to the rendering stage.

#### 3.3.2. Rendering Stage

We used OpenGL library to render the foveated view [30]. We provided the program with the coordinates of PTZ planar vertices and omnidirectional sphere vertices. While the omnidirectional sphere vertices are static (not change), the PTZ planar vertices tend to move dynamically based on the alignment model, target object orientation, and PTZ camera zoom value. This stage also uses the blend mask in PTZ planar mesh GLSL shader to do the alpha blending.

**Figure 5:**
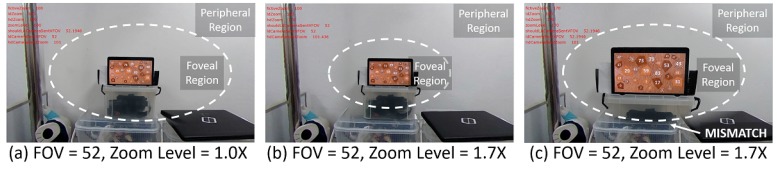
Zooming desynchronization problem. Without adjustment, the foveal region of (**a**) is getting bigger when the zoom level increased (**c**). This will induce a mismatch between foveal and peripheral region boundaries. We intended to keep the boundary seamless as shown in (**b**).

Optical zooming does not change the pixel resolution of the PTZ camera video stream, i.e., it will always be 1920 × 1080 pixels. However, the FOV of the PTZ camera is getting narrower when zooming in and wider when zooming out. These changes in FOV can cause a mismatch between the foveal and peripheral region (see Figure 5c) even though they have been previously aligned. Updating the alignment model every time the zoom level changed is infeasible due to the time overhead of the feature detection required. To solve this issue, in *Zoom-Based Adjustment* block, we adjusted the coordinates of the PTZ planar vertices based on the optical zoom value: we shrunk or enlarged the PTZ planar vertices coordinate when zoomed-in or -out respectively.

**Figure 6:**
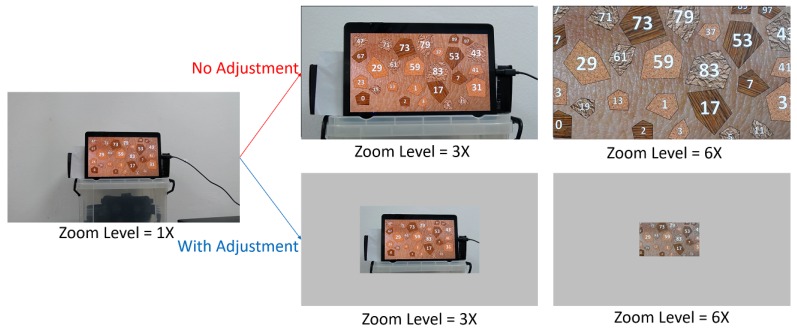
Zoom-based adjustment illustration on PTZ mesh. Without adjustment, the target object (tablet) is getting bigger when the zoom level increased. With adjustment, we shrink the PTZ vertices to preserve the target object size and position during zoom-in (as shown in this image) or enlarging the PTZ vertices during zoom-out.

Figure 6 illustrates this adjustment process. In the figure, the size of the PTZ camera image represents that of a mesh created from the PTZ vertices. Without adjustment, the mesh remains unchanged; therefore, the sizes of objects seem to get bigger or smaller based on the FOV of the PTZ camera. On the contrary, our adjustment process changes the size of the mesh in a way that keeps the sizes of objects on the image. Figure 5b shows the adjusted foveated view. Please note that the angular resolution of the foveal region is changed because of the optical zooming process. The zoom-based adjustment does not change the foveal region angular resolution any further. The adjustment only resizes the foveal region visually to keep the smoothness in peripheral and foveal region boundaries.

After adjusting the PTZ vertices based on the zoom level, we align the PTZ to the omnidirectional vertices based on the alignment model and target object orientation. We denote this process as *PTZ Vertices Alignment* in Figure 4. The remaining *Omnidirectional* and *PTZ Mesh Creation* block denote the process of UV mapping and normal vector creation.

### 3.4. Control Mechanism

Our telemedicine system has two main mechanisms: *foveal region update* and *zooming mechanism*. Unlike conventional eye tracker-based foveated rendering, our foveated view allows users to lock the location of the foveal region; therefore, they can take their eyes off from the target object and look around the space. The foveal region will be kept in high resolution until it is relocated by users through foveal region update mechanism. The zooming mechanism allows the remote physician to look closer and see the finer detail of the foveal region. These control mechanisms are the same regardless of desktop or HMD version.

#### 3.4.1. Foveal Region Update Mechanism

**Figure 7:**
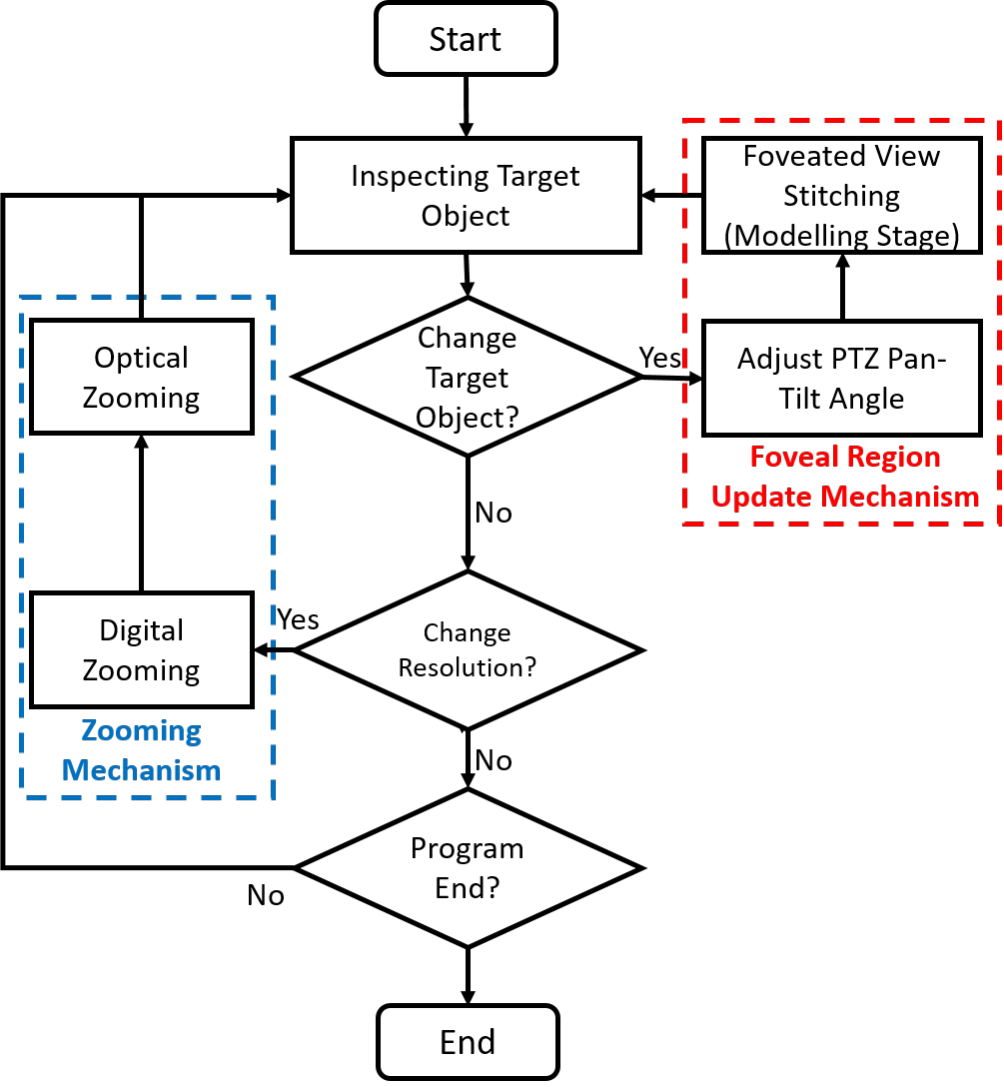
Hybrid camera control mechanism.

The overall control mechanism of our hybrid camera system is shown in Figure 7. By default, the program will allow the remote user to look around the local environment with HMD, as denoted in *inspecting target object* block. Initially, the program assumes that the target object is located in front of the PTZ camera at pan = 0${}^{\circ }$ and tilt = 0${}^{\circ }$. Thus, the foveal region will also be located at that orientation to cover the target object.

For every frame, the program will check whether the remote physician wants to change target objects or not. If s/he wants to change the target object, then s/he will rotate his/her head to focus that object in the center of his/her view. Then, the program will start the *adjust PTZ pan-tilt angle* block to point to that object. Right after the PTZ camera points to the new target object, the program will do *foveated imaging pipeline (modeling stage)* such that the foveal region will cover the new target object.

Please note that this implies the PTZ camera does not always follows the remote physician’s HMD orientation. The PTZ camera does not keep rotating in real time. Instead, it will only rotate when the remote physician intends to inspect a new object. If s/he wants to inspect a different object then the PTZ camera will rotate toward the object and the modeling stage will occur. On the contrary, if s/he only wants to have a quick look around to other objects or to communicate with local physicians then the PTZ camera will keep pointing at the previous target object. The foveal region will also not be updated. Here, we assume that the remote physician does not need to have a high resolution every time: the remote physician only needs it when necessary.

#### 3.4.2. Zooming Mechanism

**Figure 8:**
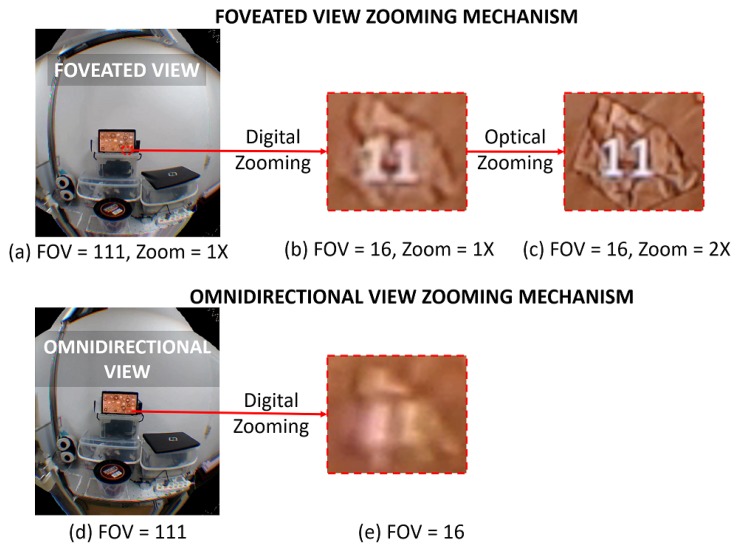
Foveated view at initial FOV and zoom level (**a**) undergoes digital (**b**) and optical zooming (**c**). The digital zooming will enlarge the target object (pentagon no. “11”) size in the view without increasing the image resolution. The follow-up optical zoom will increase image angular resolution. The omnidirectional view at initial FOV (**d**) along with its digital zoom version (**e**) is shown as a comparison. Please note that (**b**) has a higher resolution than (**e**) because (**b**) is the zooming version of the foveal region whose resolution is higher than the overall omnidirectional view.

In our zooming mechanism, we decouple the digital and optical zooming process. Each zoom occurs separately and independently from each other. We perform digital zoom by changing the FOV of the foveated view and optical zoom by changing the optical zoom parameter of the PTZ camera. Therefore, the optical zoom parameters of the PTZ camera will not be affected by the digital zoom process and vice versa. Figure 8 illustrates our decoupled zooming process. The digital zooming is denoted by the “FOV” value while optical zooming is denoted by the “ZOOM” value in the captured images in the figure. Figure 8a shows the foveated view without digital and optical zooming. If a user intends to inspect the number on the bottom right of the tablet, then s/he can zoom in by changing the FOV value to “16” (Figure 8b). However, this change will not increase the foveal region angular resolution. The number still looks blurry. Then, s/he can increase the ZOOM value to 2× to see the number more clearly—the number “5” and “11” look clearer as shown in Figure 8c.

In a practical scenario, a remote physician will first perform digital zooming. This will change the scene appearance from Figure 8a to Figure 8b. S/he can see the target object closer, but it might not be clear enough. Then s/he will perform optical zooming to increase the angular resolution. This will change the scene appearance from Figure 8b to Figure 8c. The benefit of this scenario is that the remote physician can quickly see the object closer (via digital zoom) without having to synchronize the PTZ camera optical zoom. Moreover, most PTZ cameras take time to change their optical zoom levels, thus making prompt optical-digital sync difficult. As a side note, Figure 8d,e shows the zooming process in omnidirectional view. The omnidirectional view only supports digital zooming. By comparing Figure 8d,a, the omnidirectional and foveated view seems indistinguishable. However, from Figure 8c,e, we can see that the quality of omnidirectional view is much worse than the foveated view when zoomed-in: The number “5” and “11” are indiscernible in Figure 8e, while recognizable in Figure 8c.

## 4. Evaluation and Discussion

In this section, we present evaluations of the proposed system and discuss the results and potential improvements. We first discuss the approximated angular resolution of our foveated view in comparison with human visual acuity. Secondly, we show our computation times for the modeling and rendering stages, which gives support for the separation of the two. Then we identify the two main error sources in the foveated view pipeline: pixel registration error and zoom-based adjustment error. Lastly, we provide our experimental results of the foveated view image sharpness in various zoom levels and discuss the implications.

For the experiments, the hybrid camera module and HTC Vive VR devices were connected to a single desktop PC as our focus was not on the network latency. Specifications of the desktop PC were as follows: Intel i7-7700K CPU, 16 GB RAM, 64-bit Windows 10 Pro. We used OpenFrameworks 0.10 to access hardware devices (hybrid camera module and HTC Vive VR devices) and OpenGL rendering functions. We also used OpenCV 4.0 to implement image processing algorithms.

### 4.1. Angular Resolution Approximation

We used Clark’s approach [31] to approximate human eye angular resolution. We used visual acuity of 1.7 ≈0.6arcmin/linepair. Two pixels are required to create one line pair. Thus, we approximate the human eye angular resolution for each horizontal and vertical direction as follows:

(1) $$EyeRes=60\;arcmin{/}^{\circ }\times \frac{1}{0.6}\;linepair/arcmin\times 2\;pixel/linepair=200\;pixel{/}^{\circ }$$

The maximum angular resolution that can be given by our foveated view comes from maximum foveal region angular resolution, which depends on PTZ camera video pixel size at the highest optical zoom level. First, we used the following function to find the corresponding FOV angle for certain “X×” zoom value:

(2) $$\theta_{2}=2\arctan\left(\frac{\tan\left(\frac{\theta_{1}}{2}\right)}{X}\right)$$

where $\theta_{1}$ and $\theta_{2}$ is the FOV at 1× and target X× respectively. Our PTZ camera can give up to 10× optical zoom. From Section 3.1, we get 82.15${}^{\circ }$ and 52.24${}^{\circ }$ as PTZ camera HFOV and VFOV at 1× zoom value, respectively. Inserting these values to Equation (2) resulted in 9.96${}^{\circ }$ and 5.61${}^{\circ }$ as PTZ camera HFOV and VFOV at 10× zoom value, respectively. Therefore, the PTZ camera will have a maximum horizontal and vertical angular resolution:

(3) $$HorizontalRes=1920\;pixel\times \frac{1}{9.96^{\circ }}\approx 192\;pixel{/}^{\circ }$$

(4) $$VertRes=1080\;pixel\times \frac{1}{5.61^{\circ }}\approx 192\;pixel{/}^{\circ }$$

As can be seen from Equations (3) and (4), our PTZ camera at maximum zooming value has similar angular resolution to human eye.

### 4.2. Computation Time

To measure the processing time of our foveated view stitching pipeline, we ran an image stitching process for 100 times: we used 5 levels of pan and tilt angles respectively, and 4 zoom levels per each pair of the pan-tilt angles. Table 2 shows the average processing time for each block. Preprocessing in the table refers to the process required to initialize OpenCV variables. The total computation time of the modeling stage is around 2.18 s, of which about 89% of the time was devoted to the feature detection block. The fact that we neither did limit the number of features to be detected in each camera image nor accelerated the detection algorithm with GPU in the current implementation gives a hint to a potential speed-up of the modeling stage. For example, Bian et al. [32] demonstrated a GPU accelerated detection algorithm, based on SIFT and FLANN matcher similar to ours, could detect 1000 features in tens of milliseconds.

However, a faster modeling stage would have little or no effect on the rendering stage due to the separation between the two stages in our stitching pipeline; yet, it might improve user experience. In the rendering stage, the zoom-based adjustment takes about 3 ms and the PTZ vertices alignment takes 0.01 ms. Compared to a baseline performance where we rendered the two videos without stitching, those additional processing time (3.01 ms) in the foveated view rendering caused a small decrease in frame rate: from 18 to 17 fps. In other words, users received constant foveated view updates, even with the relatively slow modeling stage. However, if they relocate the foveal region, they would need to wait for around 2.18 s to finally be able to see the target object in high-resolution. In this sense, a faster modeling stage can bring more responsiveness in the system.

### 4.3. Pixel Registration Error and Zoom-Based Adjustment Error

One way to consider the quality of our foveated view would be how well the PTZ camera image is superimposed onto the omnidirectional camera image. However, to the best of our knowledge, there are no standardized methods to measure such quality of the foveated view, specifically for a case where one of the stitched images is in equirectangular form. Therefore, we devised proxy measures that are closely related to the final quality of our foveated view: pixel registration error and zoom-based adjustment error.

Pixel registration error, $E_{pixelRegistration}$, was designed to measure the mismatch between the detected features on the omnidirectional image and the corresponding features on the PTZ image. A smaller error can lead to a better 3D alignment mapping, thus affecting the final image quality. Although Zhao et al. [33] proposed a method to find matched features between planar and omnidirectional equirectangular images, their feature detection only occurred in the equatorial region of the equirectangular image. On the other hand, in our case, users could need to locate the foveal region, i.e., PTZ image, outside the equatorial region. Thus, we decided to use a rectilinear projection for the omnidirectional image to run the SIFT feature detector on the projected planar image. Given the detected features on the omnidirectional image, $o_{n}$, and the corresponding features on the aligned PTZ image, $Hp_{n}$, we used root-mean-square error to calculate the average mismatch:

(5) $$\begin{array}{c} E_{pixelRegistration}=\sqrt{\frac{\sum_{n=0}^{N-1}{\left(o_{n}-Hp_{n}\right)}^{2}}{N-1}} \end{array}$$

We calculated the pixel registration error of the 100 stitching process trials and averaged them. The average pixel registration error was 1.45 pixels. Considering that we used 1920 × 1080 resolution for both images and that one was up-sampled, the SIFT seemed to perform well; however, the measured performance might be due to the use of the planar surface as the target object (tablet). Further research on planar-equirectangular feature matching to improve either the quality of the alignment model should be carried out.

**Figure 9:**
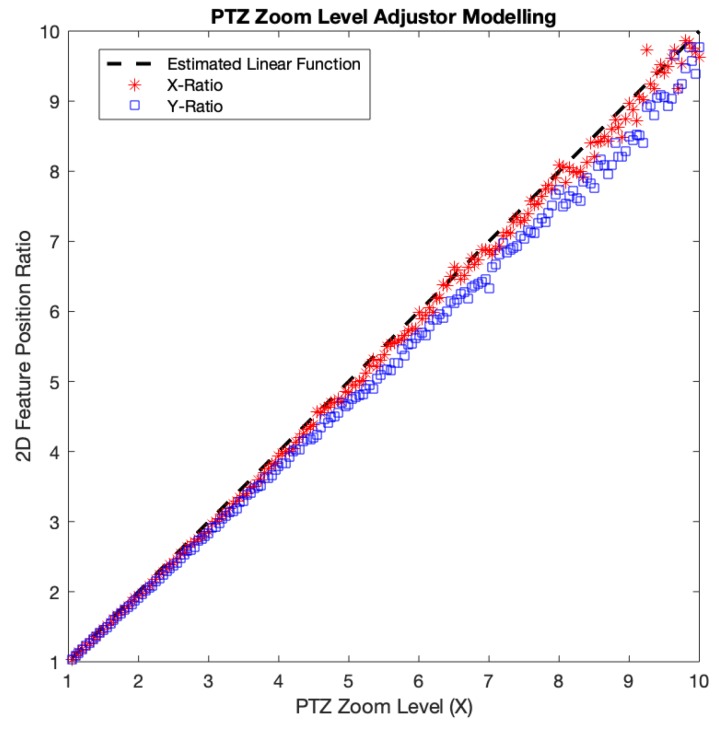
PTZ zoom-level adjustor experiment result.

*Zoom-based adjustment error* originated from our approximate linear model of the PTZ image size with regards to the PTZ zoom level in the zooming process. We chose to use the model-based approach for the optical zooming because of the time overhead of the feature detection required to adjust PTZ image size accordingly. To make the model, we measured the amount of displacement of detected SIFT features at various zoom levels from the positions of those features at the base zoom level = 1×. We incremented the PTZ camera zoom level from 1× to 10× monotonically with an interval of 0.05×. At each zoom level, we acquired the *x*-axis positions of the SIFT features and averaged their *x*-axis distances from the origin (0, 0) located at the center of the image. Then, we divided the average distance by that of the base zoom level. We did the same for the *y*-axis. Both the *x* and *y*-axis distance ratios followed the identity relation with the PTZ zoom level fairly well, with the *x* distance ratio slightly fitter (see Figure 9). Thus, for simplicity’s sake, we chose to use the identity function as our model, which in the end resulted in the zoom-based adjustment error.

The average zoom-based adjustment error per each axis was measured as 1.49% and 4.45% for *x* and *y*, respectively. In lower PTZ zoom levels, both *x* and *y*-axis distance ratios seemed to fit our model, while as the zoom level goes higher, they started to deviate. This axis-wise difference could be due to the non-linearity of PTZ camera optics as reported by Sinha et al. [17]. Also, incorrect feature matching and restricted precision in position might contribute to the error, especially when the features were located near the center of the image, which is the case for high PTZ zoom levels. The features around the origin (image center) will have small *x* and *y*-axis distances. Assuming that the features position accuracy is similar across the whole PTZ image region, the position accuracy error on each *x* and *y*-axis will have a bigger influence on features whose *x* or *y*-axis distance is small. Thus, the imprecise of feature location detection that occurred around the image center will more possibly give a higher error. Therefore, a more accurate zoom-based adjustment model built with consideration of the camera optics as well as the locations of features would possibly improve the quality of the final stitched image.

### 4.4. Image Sharpness Measure Experiment

Image sharpness determines how much detail an image can reproduce, which relates one of the goals of our foveated imaging system. We perform an experiment to measure the sharpness improvement we get from our foveated image compared with the reference omnidirectional view. We measured the Modulation Transfer Function (MTF) curve for the foveal region of the foveated images with optical zoom level 1×, 2×, 3×, ..., and 10×. We also measured the MTF curve for the omnidirectional image as the reference.

**Figure 10:**
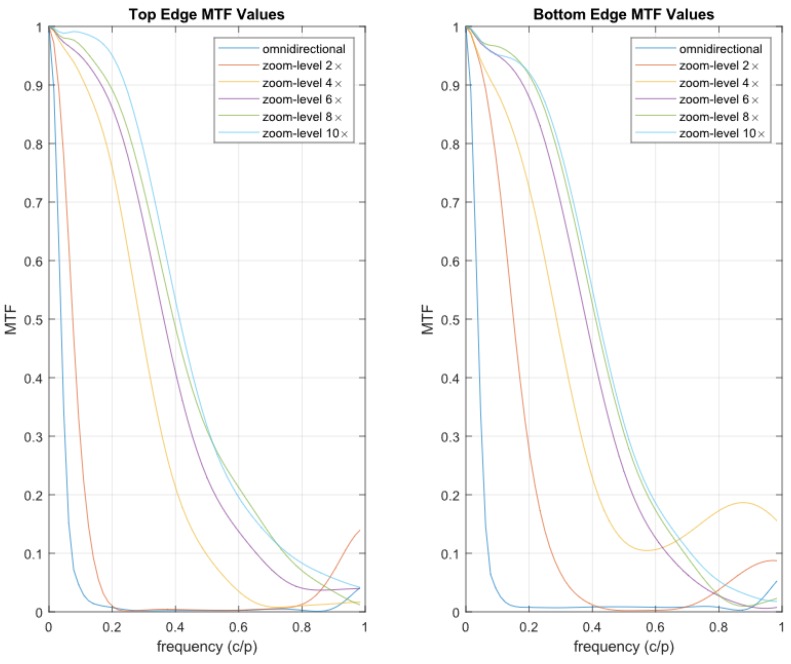
MTF Curve Result.

We used an open-source software, MTF Mapper [34], to measure the MTF. We performed a slanted-edge method. Due to the small FOV caused by 10× zoom, we used a single quadrilateral printed on a white paper, instead of the chart consisting of multiple quadrilaterals. In addition, we made the quadrilateral always placed within the foveal area regardless of the zoom level. The distance between our camera module and the paper was 1 *m*. We selected the top and bottom edges of the quadrilateral in both foveated and omnidirectional images across various zoom levels to compare. Figure 10 shows the MTF curves of even zoom levels for two edges (denoted by “top” and “bottom”) of the quadrilateral. Horizontal axis denotes frequency cycles/pixel (c/p) while vertical axis denotes MTF value, which is comparison of contrast value at frequency *f* to frequency 0. We also measured the *MTF50* (The frequency where the MTF value drop to 0.5), presented on Table 3.

Figure 10 shows that the high-frequency component (correspond to the high *x*-axis value) of the MTF curve on the foveated image tends to be higher than that of the omnidirectional image. This means that the foveated image can preserve high-resolution details better than the omnidirectional image. Table 3 also shows a similar result that the foveated image’s MTF50 is higher than that of the omnidirectional image, thus better at preserving high-resolution details.

Though in certain frequency the MTF curve seems increasing again we suspect this is because the lens is not focused on the inspected edge. During the experiment, we used autofocus features from the PTZ camera. Thus, the PTZ camera is not always focused on the edges that we inspected.

Another observation from the MTF curve of the foveated images that the curves started to saturate from the zoom level 8×. There seems no noticeable change in the MTF curve between the zoom level 8× to zoom level 10×. This is probably due to optical aberrations occurred particularly at near the extremes of their range [35]. This result implies that our proposed system no longer produces the sharpness improvement by optical zooming beyond a specific zoom level. Considering our pipeline design, this is due to the limitations of the PTZ camera we used.

## 5. Conclusions

In this paper, we presented a foveated imaging pipeline that integrates 360${}^{\circ }$ panoramic image from an omnidirectional camera with high-resolution ROI images from a PTZ camera. To make the best use of the 360${}^{\circ }$ panoramic view, we chose VR HMDs as our target viewing devices and designed the pipeline accordingly. The proposed pipeline consisted of two stages: modeling and rendering, and the parallel executions of the two stages allowed our system to achieve a high frame rate of the foveated view, which is required for the use of VR HMDs. The use of the PTZ camera made it easy to relocate the foveal region, and also made our system capable of zooming on the foveated view, which guarantees the usefulness of our pipeline even when used a viewing device with lower resolution. In addition, our hybrid camera module can potentially be integrated with other telemedicine research to provide better video quality on the local environment.

From the experiment, we showed that the foveated view can roughly provide the remote physician with a view whose angular resolution similar to human visual acuity. We also showed that the separation of the modeling from the rendering stage allows the application to render the foveated view near real time. Precisely, even though the modeling stage required around 2.18 s to update the alignment model, the system can still run in 17 fps. The proposed pipeline also showed good quality by having only 1.45 pixels registration error and 1.49% and 4.45% for *x* and *y*-coordinate ratio relative error, respectively. From the MTF score, we showed that the separation of the omnidirectional sphere and PTZ planar mesh allowed the foveated view to preserve the high-resolution component of images better than the omnidirectional view while zooming in.

Future work should focus on improving the performance of our proposed system. For example, we assumed, in a medical scenario, the remote physician might not need to update the ROI frequently, so the delay caused by updating the model would not degrade the usability of our system on a scale. However, if our system is applied to a scenario where a remote user keeps changing the foveal region, the user might get frustrated from the delayed reflection of his/her intention. Clever use of extrinsic parameters in the modeling stage [19] or GPU acceleration would speed up the feature detection. In relation to that, planar-equirectangular image feature detector also should be further investigated. In addition, supporting motion parallax would greatly improve user experience. Though a motorized platform can achieve this, a deep-learning model-based view synthesis might also be used to address this issue.

Although we targeted telemedicine, our proposed system can also be used in other applications. In remote teleoperation, the capability of rendering high angular resolution on the VR HMD will allow the remote operator to control the local mobile robot better and precisely. For example, in a search and rescue task [36], the use of immersive HMD will improve the situation awareness in the disaster site for the remote operator. The high resolution of the foveal region will also help him to locate victims. In addition, remote collaboration can also benefit from our 360${}^{\circ }$ high-resolution foveated image. Recently researchers have attempted to use a 36${}^{\circ }$ camera as an alternative of a 3D reconstructed virtual space or a live 2D image-based mixed reality collaboration [37]. In such a collaboration situation, the remote user’s capability to view high-resolution in 360-degree can allow the remote participant to inspect the far located object without having to ask the local participant to carry the camera. With the proliferation of the omnidirectional camera and VR HMD, we believe our proposed foveated imaging system can be useful in various fields.

## Figures and Tables

**Table 1:** Comparison with Previous Works.

Methods	Cameras Used	Output View	Stitching Method
Qin et al. [15]	Wide-FOV and High-Res	Separated 2D Views	No Stitching
Sinha et al. [17]	PTZ	3D Cubemap	3D Reconstruction
Lin et al. [18]	Wide-FOV and PTZ	2D Multi-Res Wide-FOV Display	Pixel-Level Stitch
Dornaika et al. [19]	Wide-FOV and High-Res	2D Multi-Res Wide-FOV	Pixel-Level Stitch
Chen et al. [20]	Wide-FOV and PTZ	2D Projector Screen	Superimposing
Lee et al. [22]	High-Res Camera Arrays	Foveated 360${}^{\circ }$ Image	Pixel-Level Stitch
Lee et al. [23]	High-Res Camera Arrays	Foveated 360${}^{\circ }$ Image	Pixel-Level Stitch
Ours	360${}^{\circ }$ and PTZ	Zoomable Foveated 360${}^{\circ }$ Image	Vertice-Level Stitch

**Table 2:** Foveated imaging pipeline modeling stage computational time.

Feature Detection				Feature Matching		Image Alignment		Preprocessing		Total	
Theta		PTZ									
sec	%	sec	%	sec	%	sec	%	sec	%	sec	%
1	49	0.8	40	0.1	4	0.001	0.04	0.28	6.96	2.18	100

**Table 3:** MTF50 results.

Edge	Omnidirectional	1×	2×	3×	4×	5×	6×	7×	8×	9×	10×
	(c/p)	(c/p)	(c/p)	(c/p)	(c/p)	(c/p)	(c/p)	(c/p)	(c/p)	(c/p)	(c/p)
Top	0.045	0.079	0.145	0.227	0.274	0.313	0.339	0.352	0.359	0.357	0.373
Bottom	0.044	0.073	0.144	0.22	0.265	0.338	0.352	0.373	0.372	0.382	0.371
